# Supplementary material for: Scalable Boltzmann generators for equilibrium sampling of large-scale materials
Source: Nat Commun. 2026 Jun 5;17:5010. doi: 10.1038/s41467-026-73900-9 (PMC13241520; doi:10.1038/s41467-026-73900-9)
Supplement: Supplementary file 1 — Supplementary Information [file 41467_2026_73900_MOESM1_ESM.pdf]

Supplementary Information for 'Scalable Boltzmann Generators for equilibrium sampling of large-scale materials'

Maximilian Schebek[1,*], **Frank Noé**[1,2,3,4], **Jutta Rogal**[1,5]

[1] Fachbereich Physik, Freie Universität Berlin, 14195 Berlin, Germany
[2] Fachbereich Mathematik und Informatik, Freie Universität Berlin, 14195 Berlin, Germany
[3] Microsoft Research AI for Science, 10178 Berlin, Germany
[4] Department of Chemistry, Rice University, Houston, Texas 77005, USA
[5] Initiative for Computational Catalysis, Flatiron Institute, New York, New York 10010, USA
[*] Author to whom any correspondence should be addressed.

E-mail: `m.schebek@fu-berlin.de`

11 May 2026

1. Augmented variables

1.1. Limitations of standard coupling flows for local environments

Coupling flows [1, 2] enable efficient density estimation by partitioning the input coordinates into two channels, $\mathbf{x} = (\mathbf{x}_A, \mathbf{x}_B)$, and transforming one channel conditioned on the other according to $\mathbf{x}'_A = g(\mathbf{x}_A \mid C(\mathbf{x}_B))$ while $\mathbf{x}'_B = \mathbf{x}_B$. Here, g is a bijective transformation and C is a conditioner. However, while this partitioning allows for efficient density estimation, it introduces a structural limitation when modeling atomistic systems based on local environments. In atomistic models, physically relevant features typically depend on full three-dimensional relative coordinates between particles, i.e., $\mathbf{r}_{ij} = \mathbf{x}_i - \mathbf{x}_j$. These quantities require simultaneous access to all Cartesian components of the particle coordinates.

This limitation can already be illustrated using a two-particle system with coordinates $\mathbf{x} = (\mathbf{x}_1, \mathbf{x}_2)$ and $\mathbf{x}_i \in \mathbb{R}^3$. Consider a coupling flow that partitions the system such that $\mathbf{x}_A = \mathbf{x}_1$ and $\mathbf{x}_B = \mathbf{x}_2$. The conditioner $C(\mathbf{x}_B)$ then only has access to $\mathbf{x}_2$, but, since $\mathbf{x}_1$ is part of the transformed channel, the conditioner does not have access to $\mathbf{r}_{12}$, preventing transformations that depend on complete pairwise environments. Augmented flows can overcome these limitations and can compute difference vectors and distances within physical and auxiliary spaces separately as discussed in the main text (see also Sec. 3).

1.2. Free energy

In the following, we show that the partition function of the joint system as defined in the main text factorizes, resulting in an additive total free energy. Consider a distribution on the augmented space of the form

$$\mu(\mathbf{x}, \mathbf{a}) = \frac{e^{-u_\mu(\mathbf{x})}\, e^{-(\mathbf{a}-\mathbf{x})^2/2\eta_\mu^2}}{Z_\mu^{\mathrm{aug}}} \quad . \tag{1}$$

In this case, the partition function of the augmented system can be written as

$$Z_\mu^{\mathrm{aug}} = \iint d\mathbf{x} d\mathbf{a}\, \mu(\mathbf{x}, \mathbf{a}) = \int d\mathbf{x}\, e^{-u_\mu(\mathbf{x})} \int d\mathbf{a}\, e^{-(\mathbf{a}-\mathbf{x})^2/2\eta^2} \tag{2}$$

$$= \int d\mathbf{x}\, e^{-u_\mu(\mathbf{x})} Z_\mu^{\mathrm{aux}}(\mathbf{x}) = Z_\mu^{\mathrm{aux}} \int d\mathbf{x}\, e^{-u_\mu(\mathbf{x})} \tag{3}$$

$$= Z_\mu \cdot Z_\mu^{\mathrm{aux}} \quad , \tag{4}$$

where $Z_\mu = \int d\mathbf{x}\, e^{-u_\mu(\mathbf{x})}$ and $Z_\mu^{\mathrm{aux}}(\mathbf{x}) = \int d\mathbf{a}\, e^{-(\mathbf{a}-\mathbf{x})^2/2\eta_\mu^2}$ are the partition functions of physical and auxiliary systems, respectively. Since the integral over a normal distribution is independent of its mean, $Z_\mu^{\mathrm{aux}}(\mathbf{x})$ is independent of $\mathbf{x}$ and can be pulled out of the integral in Supplementary Eq. (3). It follows immediately that

$$f_\mu^{\mathrm{aug}} = -\log Z_\mu^{\mathrm{aug}} = -\log Z_\mu - \log Z_\mu^{\mathrm{aux}} = f_\mu + f_\mu^{\mathrm{aux}} \quad . \tag{5}$$

1.3. Constraining the center of mass

An important property of the potential energy in many-body systems is its invariance under global translations of the system. Therefore, optimizing the flow parameters without constraints could lead to an uncontrolled motion of the center of mass, distorting the free energy estimates [3, 4]. To prevent this, we follow Supplementary Reference [5] and directly model the physical coordinates $\mathbf{x}$ on the mean-free space $\mathbb{R}^{3N}/\mathbb{R}^3$, which is achieved by swapping the center of mass between physical and auxiliary systems using a ShiftCoM layer after each coupling layer [5]. The ShiftCOM layer applies the transformation

$$(\mathbf{x}, \mathbf{a}) \mapsto (\mathbf{x} - \bar{\mathbf{a}}, \mathbf{a} - \bar{\mathbf{a}}), \tag{6}$$

where $\bar{\mathbf{a}} = \frac{1}{N}\sum_{i=1}^{N} \bar{\mathbf{a}}_i$. Interleaving the ShiftCoM layer with the coupling layers temporarily removes the center-of-mass (CoM) constraint on the physical variables, while ensuring that the final transformation maps onto the mean-free subspace. See Supplementary Reference [5] for details.

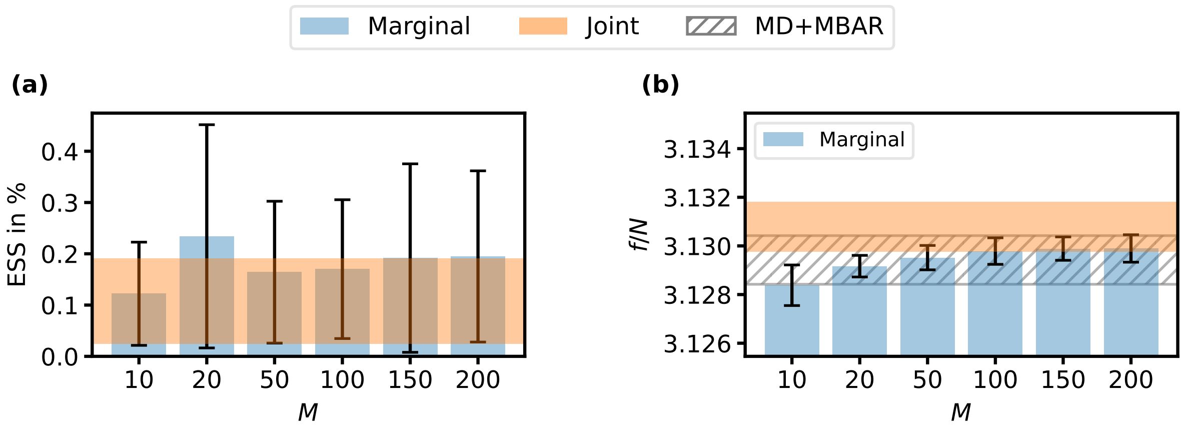

Supplementary Figure 1: Convergence of the ESS (**a**) and the free energy estimate (**b**), as obtained from marginal (blue) and joint (orange) densities, with respect to the number of auxiliary samples M drawn per generated physical sample for the FCC Lennard–Jones system with $N = 864$. Error bars were estimated over four independently trained models, each evaluated three times.

1.4. Convergence of the marginal distribution

As discussed in the main text, the marginal distribution can be estimated using

$$q_\theta(\mathbf{x}') \approx \frac{1}{M} \sum_{m=1}^{M} \frac{q_\theta(\mathbf{x}', \mathbf{a}_m)}{\pi(\mathbf{a}_m|\mathbf{x}')} \quad , \tag{7}$$

where M is the number of auxiliary samples drawn per generated physical sample. Figure 1 shows the convergence of the ESS (left) and the free energy estimate (right), both obtained from the marginal density, as a function of M for the FCC Lennard–Jones system with $N = 864$. Importantly, while the ESS is independent of M, the free energy estimate converges around $M = 100$.

2. Systems

2.1. Lennard-Jones

The pairwise LJ potential is given by [6]

$$u(r) = 4\epsilon \left[\left(\frac{\sigma}{r}\right)^{12} - \left(\frac{\sigma}{r}\right)^{6} \right], \tag{8}$$

where r is the two-particle distance. ε and σ define the characteristic length and energy scales, respectively. A cutoff radius r_{cut} is typically employed and the potential is shifted to be continuous at the cutoff. This results in the following interaction potential:

$$u_{\text{cut}}(r) = \begin{cases} u(r) - u(r_{\text{cut}}) & \text{if } r \leq r_{\text{cut}}, \\ 0 & \text{else.} \end{cases} \tag{9}$$

To simplify comparisons between different systems, reduced units based on ε and σ are commonly used, and quantities in these units are typically denoted by an asterisk.

2.2. Stillinger-Weber

The SW potential [7] features two-body (ϕ_2) and three-body (ϕ_3) interactions, of which the latter enforce tetrahedral coordinations. The total potential energy is given by

$$U_{\mathrm{SW}}(\mathbf{x}) = \sum_i \sum_{j>i} \phi_2(d_{ij}) + \lambda_3 \sum_i \sum_{j\neq i} \sum_{k>j} \phi_3(d_{ij}, d_{ik}, \theta_{ijk}) \quad , \tag{10}$$

with

$$\phi_2(r) = A\epsilon \left[B \left(\frac{\sigma}{r}\right)^4 - 1 \right] \exp\left(\frac{\sigma}{r - a\sigma}\right) \quad , \tag{11}$$

$$\phi_3(r, s, \theta) = \lambda\varepsilon(\cos\theta - \cos\theta_0)^2 \exp\left(\frac{\gamma\sigma}{r - a\sigma}\right) \exp\left(\frac{\gamma\sigma}{s - a\sigma}\right), \tag{12}$$

where d_{ij} denotes a two-particle distance and θ_{ijk} is the angle formed by the three atoms. All parameters are fixed except for ε, σ, and λ_3, which are adjusted to model a specific system. Here, λ_3 sets the strength of the three-body interactions, while ε and σ define the characteristic energy and length scales. The common constants are $A = 7.049556277$, $B = 0.6022245584$, $a = 1.8$, $\theta = 109.47°$, and $\gamma = 1.2$. For the monatomic water (mW) model, the tuned values are $\lambda = 23.15$, $\varepsilon = 6.189$ kcal/mol, and $\sigma = 2.3925$ Å [8], while for silicon $\lambda = 21$, $\varepsilon = 50.003$ kcal/mol, and $\sigma = 2.0951$ Å. As in the Lennard–Jones potential, reduced units can be adopted in which ε and σ serve as the energy and length scales. In this representation, the only parameter that differentiates models is λ_3.

2.3. Unit cells

Table 1 summarizes the unit cells used for the different crystal structures.

Supplementary Table 1: Unit cell shapes and number of atoms for different crystal structures.

Crystal Structure	Unit Cell Shape	Number of Atoms per Unit Cell
cubic ice	cubic	8
diamond cubic	cubic	8
hexagonal ice	orthorhombic	8
FCC	(cubic / orthorhombic)	(4 / 6)
HCP	orthorhombic	4
BCC	cubic	2
β-tin	orthorhombic	4

In the context of computing the free energies of the different phases in the Stillinger-Weber potential as a function of the three body interaction (Fig. 6 in the main text), we observed that the β-tin phase exhibits subtle structural modifications as a function of the three-body interaction strength. In particular, we found that the equilibrium lattice can be written as

$$\mathbf{R} = \mathbf{R}_0 + \delta_{\beta\text{-tin}}(\lambda_3)\boldsymbol{\Delta} \quad , \tag{13}$$

where $\mathbf{R}_0$ represents the ideal β-tin unit cell and $\boldsymbol{\Delta}$ is a perturbation matrix:

$$\mathbf{R}_0 = \begin{bmatrix} 0.0 & 0.0 & 0.0 \\ 0.0 & 0.5 & 0.25 \\ 0.5 & 0.5 & 0.5 \\ 0.5 & 0.0 & 0.75 \end{bmatrix} , \quad \boldsymbol{\Delta} = \begin{bmatrix} 0.0 & 0.0 & 0.0 \\ 1.0 & 0.0 & 0.0 \\ 1.0 & 1.0 & 0.0 \\ 0.0 & 1.0 & 0.0 \end{bmatrix} \quad . \tag{14}$$

To improve numerical convergence, we set $\delta_{\beta\text{-tin}} = 0.08$ for the initialization of the base distribution which resulted in significantly more stable optimization compared to the ideal case ($\delta_{\beta\text{-tin}} = 0.0$). For the calculation of the Silicon phase diagram (Fig. 7 in the main text), we set $\delta_{\beta\text{-tin}} = 0.0$.

3. Model details

3.1. Graph neural network

The graph neural network used for computing the particle embeddings is composed of L layers, where the update of the lth layer is calculated as

$$\begin{aligned} \mathbf{d}_{ij} &= \text{sinusoidal}([\mathbf{a}_i - \mathbf{a}_j]_{\text{PBC}}; \boldsymbol{\omega}_d) \quad , \\ \mathbf{m}_{ij}^l &= \phi_e(\mathbf{h}_i^l, \mathbf{h}_j^l, \mathbf{d}_{ij}) \quad , \\ \mathbf{m}_i^l &= \sum_{j \in \mathcal{N}_i} \mathbf{m}_{ij}^l \quad , \\ \mathbf{h}_i^{\mathbf{a},l+1} &= \phi_h(\mathbf{h}_i^{\mathbf{a},l}, \mathbf{m}_i^l) \quad . \end{aligned} \tag{15}$$

$\mathcal{N}_i$ contains all particles defined as the local neighborhood around the auxiliary particle i and ϕ_e and ϕ_h are implemented as multilayer perceptrons (MLP). The notation $[\cdot]_{\text{PBC}}$ indicates that the difference vector is to be computed respecting periodic boundary conditions. To increase expressivity, we make use of sinusoidal embeddings (see following subsection) for both the difference vectors and the initial node embedding, using separate base frequencies $\boldsymbol{\omega} \in \mathbb{R}^3$ for each.

3.2. Sinusoidal encoding

We use sinusoidal encodings to encode difference vectors and ideal lattice positions. Initial embeddings are set as $\mathbf{h}_i^0 = \text{sinusoidal}(\mathbf{x}_i^0; \boldsymbol{\omega}_{\text{init}})$, where $\mathbf{x}_i^0$ is the ideal lattice

positions of atom i. We set $\boldsymbol{\omega}_{\text{init}}$ to match the unit cell, so atoms in repeating cells share embeddings. Longer frequencies that uniquely label each particle perform similarly, but the unit-cell-based approach is naturally transferable to any supercell. For the sinusoidal encoding of dimension j of a vector $\mathbf{x} \in \mathbb{R}^3$, we use [4]

$$\begin{aligned} \text{sinusoidal}(\mathbf{x}, \boldsymbol{\omega})_j = [\, & \cos(\omega_j x_j) \quad, \sin(\omega_j x_j), \\ & \cos(2\omega_j x_j), \sin(2\omega_j x_j) \quad, \\ & \ldots, \\ & \cos(N_f \omega_j x_j), \sin(N_f \omega_j x_j] \quad, \end{aligned} \tag{16}$$

where N_f is the total number of frequencies and $\boldsymbol{\omega} \in \mathbb{R}^3$ contains the base frequencies for the three spatial dimensions.

3.3. Conditional training

Conditioning on external parameters such as the shape of the simulation box, parameters of the interaction potential, or thermodynamic states, is achieved by minimizing the conditional loss function

$$\mathcal{L}_{qp}(\theta) = -\mathbb{E}_{\mathbf{c}\sim p_{\mathbf{c}}} \mathbb{E}_{\mathbf{x}\sim q} \big[\log w(\mathbf{x}|\mathbf{c})\big] \quad , \tag{17}$$

where $p_{\mathbf{c}}$ defines the distribution of the conditional parameter $\mathbf{c}$. If no prior information about the system's shape distribution is available, a reasonable estimate can be obtained from short MD simulations at very small system sizes.

As an example, Supplementary Figure 2 shows the free-energy performance of a BG trained with volume conditioning for cubic silicon modeled with the SW potential. The BG produces highly accurate estimates across all densities considered. This density range corresponds to volumes that occur in the NPT simulations used to compute the silicon phase diagram in the main text.

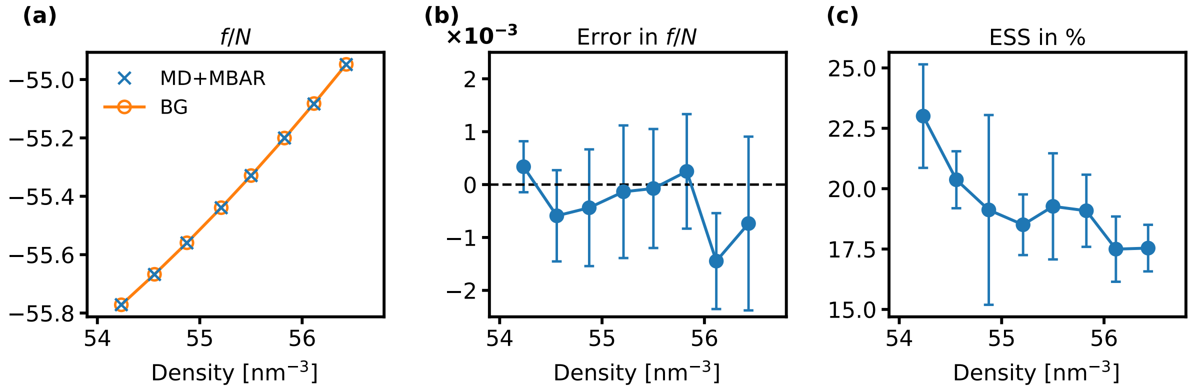

Supplementary Figure 2: **a** Reduced absolute Helmholtz free energies per particle of cubic silicon in the SW potential as a function of particle density at $T = 785\,\mathrm{K}$, obtained from MD+MBAR and from the volume-conditioned BG. **b** Deviations of the BG predictions from MD+MBAR. **c** Effective sample size. Error bars were estimated from five independent evaluations of a single model.

3.4. Hyperparameters

Supplementary Table 2: Model hyperparameters of the local augmented flow.

**Normalizing flow**	
Number of layers	10 (mW/SW), 8 (LJ)
**Graph neural network**	
Number of layers	2
Embedding dimension	96
Number of frequencies in sinusoidal embedding (N_f)	8
Number of neighbors for message passing ($\mathcal{N}_i$)	16 (cubic ice, diamond cubic), 17 (hexagonal ice), 12 (HCP and FCC LJ, 14 (BCC), 16 (β-tin)
**Circular rational-quadratic spline**	
Number of segments	16
**Base distribution**	
η_q	0.05σ (LJ), 0.2 Å(mW/SW)

Supplementary Table 3: Model hyperparameters of the global flow.

**Normalizing flow**	
Number of layers	12
**Transformer**	
Number of blocks	2
Number of heads	2
Embedding dimension	128
Number of frequencies in sinusoidal encoding (N_f)	8 (mW 64 particles), 16 (mW 216 and LJ 256 particles)
**Circular rational-quadratic spline**	
Number of segments	16
**Base distribution**	
η_q	0.01σ (LJ), 0.2 Å(mW, SW)

4. Flow-based Gibbs free energy calculations

4.1. Legendre transformation

For the minimization $G = \min_{\mathbf{h}}[F(\mathbf{h}) + \det(\mathbf{h})P]$, $F(\mathbf{h})$ is evaluated using 10^3 samples, while 10^4 samples were used for a final evaluation of G at the optimal box parameteres. To reduce memory costs, $\tilde{G}(\mathbf{h})$ is minimized using gradient-free methods like Nelder–Mead [9], typically converging within a few tens of steps. Only isotropic deformations were allowed, except for β-tin, where the box was flexible in all three dimensions. For MD-based calculations, $\langle \mathbf{h} \rangle_{T,P}$ is obtained from NPT simulations and $F(\langle \mathbf{h} \rangle)$ from NVT, giving $G = F + P \det\langle \mathbf{h} \rangle_{T,P}$. The two approaches reproduce the same result when the minimizing box matches the mean observed in MD, which holds for all systems investigated and is consistent with the harmonic approximation.

4.2. Derived quantities

The Gibbs free energies computed in this manner also enable the calculation of thermodynamic response functions, as exemplified here for the isothermal compressibility. In general, response functions can be obtained from pressure derivatives of the Gibbs free energy. The volume follows from the first pressure derivative at constant temperature,

$$V = \left(\frac{\partial G}{\partial P}\right)_T, \tag{18}$$

and the isothermal compressibility from the second pressure derivative,

$$\kappa_T = -\frac{1}{V}\left(\frac{\partial V}{\partial P}\right)_T = -\frac{1}{V}\left(\frac{\partial^2 G}{\partial P^2}\right)_T. \tag{19}$$

In practice, $V(P)$ and $\kappa_T(P)$ are obtained from numerical derivatives of $G(P)$ evaluated on a discrete pressure grid. To obtain stable higher-order derivatives, the $G(P)$ data are smoothed using a local polynomial (Savitzky–Golay) filter prior to differentiation, and the resulting $V(P)$ curve is additionally fitted with an equation of state (here a Murnaghan form) to provide robust pressure derivatives.

For validation, the compressibilities obtained in this way can be compared with independent reference estimates derived from alternative free-energy calculations processed using the same analysis protocol, as well as from direct molecular dynamics simulations in the NPT ensemble. In the latter case, the isothermal compressibility is given by the fluctuation formula

$$\kappa_T = \frac{\langle V^2\rangle - \langle V\rangle^2}{k_B T\langle V\rangle}. \tag{20}$$

Figure 3 shows the resulting isothermal compressibility for cubic silicon ($N = 216$) over the pressure range 10–15 GPa. The compressibility derived from the Gibbs free energy is in good agreement with the molecular dynamics reference data, both in absolute magnitude and in its pressure dependence, including the expected decrease of κ_T with increasing pressure.

5. Free energy estimates

Fig. 4 shows the reduced absolute Helmholtz free energies per particle, while the corresponding errors are presented in Fig. 5.

6. Training and evaluation costs

Unless stated otherwise, all models were trained on NVIDIA A5000 GPUs, using four GPUs in parallel. For the mW ice system with $N = 216$, training the local flows took approximately four days for 1 million steps, while the global flows required around 2 days. For the LJ system, the local flows took about 3 days, and the global flows took around 2 days. The smaller mW system with $N = 64$ required around 2 days of training on a single GPU for the global flow and and around 3 days for the local flow. To enable a direct comparison of training costs with the results of Ref. [4, 10], we also trained one model on cubic mW ice with $N = 216$ using four NVIDIA A100 GPUs. The training required two days for one million steps, with convergence reached after 500k steps (or approximately one day).

Generating 50k samples and computing a free-energy estimate based on the joint density took around 10 minutes for the largest systems and about 1 minute for the smallest systems, while computing the marginal density increased the cost by a factor approximately equal to the number of marginal samples M.

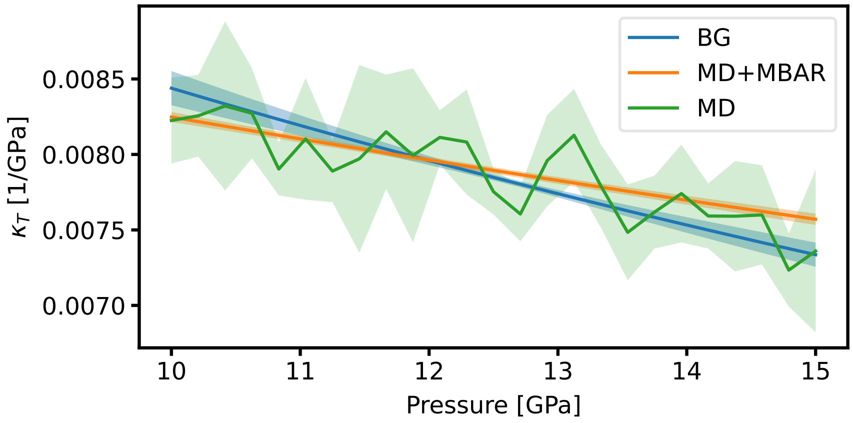

Supplementary Figure 3: Isothermal compressibility κ_T at $T = 785\,\mathrm{K}$ of cubic silicon as a function of pressure obtained from three independent estimators: derivatives of the BG-derived Gibbs free energy combined with a Murnaghan equation-of-state fit to the corresponding volume $V(P)$ (blue), derivatives of the MBAR Gibbs free energy $G(P)$ processed using the same smoothing and equation-of-state fitting procedure (orange), and volume fluctuations from MD simulations (green). Solid lines denote the mean values, while shaded bands indicate the corresponding uncertainties obtained over five runs.

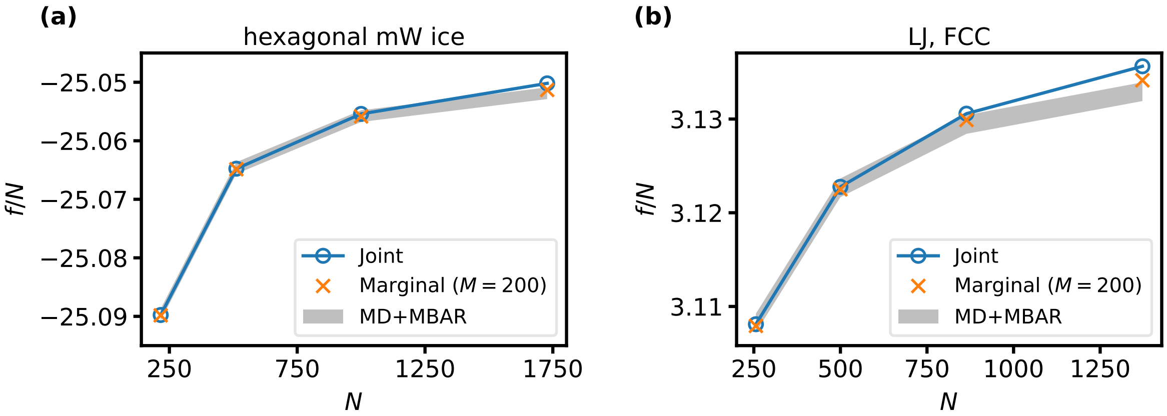

Supplementary Figure 4: Reduced absolute Helmholtz free energies per particle of hexagonal ice in the mW potential (**a**) and the FCC crystal in the LJ potential (**b**) against the number of particles.

7. Optimization details

We used the Adam optimizer [11] for the training of all models and used gradient clipping for stability. Further, the initial learning rate of 10^{-4} was reduced after 250k and 500k steps by a factor of 10. We followed Supplementary Reference [4] and trained

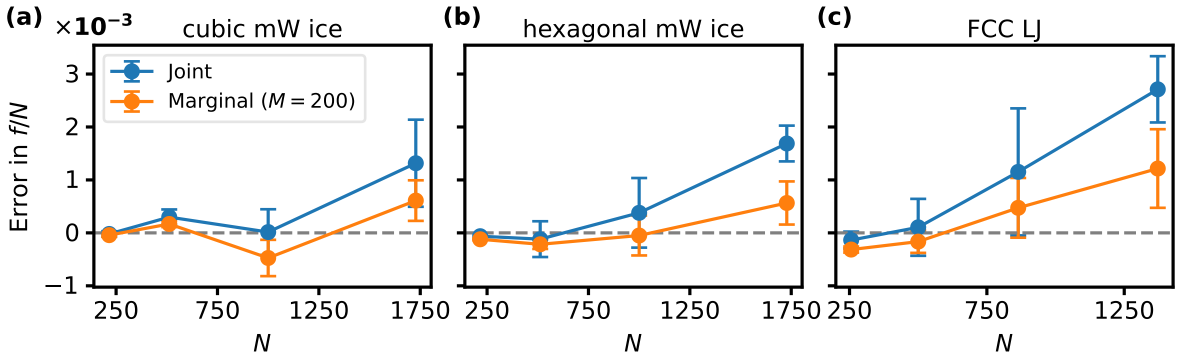

Supplementary Figure 5: Error in the reduced absolute Helmholtz free energies of cubic and hexagonal ice in the mW potential (**a** and **b**) and the FCC crystal in the LJ potential (**c**) against the number of particles. Error bars were obtained over four independently trained models each evaluated three times.

on a linearized version of the interaction potentials, defined by

$$u_{\text{lin}}(r) = \begin{cases} u(r_{\text{lin}}) + u'(r_{\text{lin}})(r - r_{\text{lin}}) & r < r_{\text{lin}} \\ u(r) & r \geq r_{\text{lin}}, \end{cases} \tag{21}$$

where u is the original pairwise potential and r_{lin} is a distance threshold below which it is linearized. Setting r_{lin} smaller than the typical distance between particles stabilizes training without biasing the target distribution.

Supplementary References

[1] L. Dinh, D. Krueger, and Y. Bengio. Nice: Non-linear independent components estimation, 2014. arXiv preprint, `https://arxiv.org/abs/1410.8516`.

[2] L. Dinh, J. Sohl-Dickstein, and S. Bengio. Density estimation using real NVP. In *International Conference on Learning Representations (ICLR)*, 2017.

[3] R. Ahmad and W. Cai. Free energy calculation of crystalline solids using normalizing flows. *Model. Simul. Mat. Sci. Eng.*, 30(6):065007, 2022.

[4] P. Wirnsberger, G. Papamakarios, B. Ibarz, S. Racanière, A. J. Ballard, A. Pritzel, and C. Blundell. Normalizing flows for atomic solids. *Mach. Learn. Sci. Technol.*, 3(2):025009, 2022.

[5] L. I. Midgley, V. Stimper, J. Antoran, E. Mathieu, B. Schölkopf, and J. M. Hernández-Lobato. SE(3) equivariant augmented coupling flows. In *Thirty-seventh Conference on Neural Information Processing Systems (NeurIPS)*, 2023.

[6] D. Frenkel and B. Smit. *Understanding Molecular Simulation: From Algorithms to Applications*, volume 1 of *Computational Science Series*. San Diego, second edition, 2002.

[7] F. H. Stillinger and T. A. Weber. Computer simulation of local order in condensed phases of silicon. *Phys. Rev. B*, 31(8):5262, 1985.

[8] V. Molinero and E. B. Moore. Water modeled as an intermediate element between carbon and silicon. *J. Phys. Chem. B*, 113(13):4008–4016, 2009.

[9] J. A. Nelder and R. Mead. A simplex method for function minimization. *Comput. J.*, 7(4):308–313, 1965.

[10] P. Wirnsberger, B. Ibarz, and G. Papamakarios. Estimating gibbs free energies via isobaric-isothermal flows. *Mach. Learn. Sci. Technol.*, 4(3):035039, 2023.

[11] D. P. Kingma and J. Ba. Adam: A method for stochastic optimization, 2014. arXiv preprint, `https://arxiv.org/abs/1412.6980`.
